# The mediating effect of coping styles between self-compassion and body image disturbance in young breast cancer survivors: a cross-sectional study

**DOI:** 10.1186/s12912-023-01342-5

**Published:** 2023-05-23

**Authors:** Fei Zhu, Chunlei Liu, Wan Zhang, Wanmin Qiang, Xiaoping Yin, Qian Lu

**Affiliations:** 1 School of Nursing, Hebei University, Baoding, Hebei Province China; 2 Tianjin Medical University Cancer Institute and Hospital, Tianjin, China; 3 Division of Medical & Surgical Nursing, School of Nursing, Peking University, #38 Xueyuan Road, Haidian District, Beijing, 100191 China

**Keywords:** Young breast cancer survivors, Coping styles, Self-compassion, Body image disturbance

## Abstract

**Background:**

Young breast cancer survivors with body image disturbance have poor quality of life. Self-compassion and different coping styles can influence their body image. The purpose of the study was to investigate the relationship between self-compassion, coping styles, and body image disturbance, and examined the mediation role of coping styles between self-compassion and body image disturbance among young breast cancer survivors in China.

**Methods:**

In the cross-sectional study, a total of 310 young women with breast cancer were assessed on self-compassion, coping styles, and body image disturbance by self-reported questionnaires in China. Spearman’s correlation was used to test the links between variables and to verify the indirect effects between variables by constructing a structural equation model.

**Results:**

There were correlations between self-compassion, different coping styles, and body image disturbance. Confrontation, avoidance, and acceptance-resignation coping had significant mediation effects on the association between self-compassion and body image disturbance. The mediation effects of confrontation coping were greater than avoidance, and acceptance-resignation coping.

**Conclusions:**

In this study, different coping styles acted as mediators between self-compassion and body image disturbance, providing support for further understanding the mechanism between self-compassion and body image disturbance, and developing comprehensive interventions for body image disturbance. Oncology nurses should pay attention to the breast cancer survivors’ self-compassion and coping styles and encourage them to adopt adaptive coping strategies, which can reduce body image disturbance.

## Background

The global incidence of breast cancer among younger adults is increasing annually [1], and more than 20% of breast cancer survivors (BCS) in China are aged below 50 years [2]. With advances in medical screening and treatment, the breast cancer 5-year survival rate has increased to 89.4% [3]. However, cancer treatment alters the physical appearance and functions of BCS (e.g., breast asymmetry, scars, alopecia, overweight, sexual dysfunction, lymphedema, etc.) [4], resulting in body image disturbance (BID) in BCS [5]. BID in cancer survivors encompasses three attributes: (1) negative self-perception concerning appearance changes and others’ evaluation of such changes; (2) physical and social function decline caused by treatment (e.g., activity limitation and social avoidance); and (3) psychological distress regarding physical appearance and function (e.g., anxiety and depression) [5]. Women with breast cancer also suffer from BID in China [6, 7]. For Chinese women, breasts are a symbol of female identity, and maintaining a traditional body image can be socially accepted and avoid others’ negative comments about the appearance [6]. On the other hand, under the influence of traditional Chinese family culture, women are expected to assume the role of mother and wife. Physical dysfunction prevents women from resuming a normal life in a timely manner and may need to be taken care of, affecting family harmony and increasing women’ psychological distress [6]. Zhou et al. [7] reported that the prevalence of BID among surveyed Chinese women who underwent breast cancer treatment was 52.5%. Compared with elderly BCS, young BCS are more likely to associate self-worth with appearance; furthermore, their BID is more severe [8, 9]. Currently, there is no consensus on the definition of the age range of young breast cancer patients. However, some relevant studies defined “young breast cancer” as women under the age of 50 years, and it was reported that dissatisfaction with the body seriously affects the quality of life of young women with breast cancer (≤ 50 years) [4, 10]; therefore, in this study, women with breast cancer aged 18–50 years were defined as young BCS. BID is influenced by several factors such as individual cognition, attitudes, and emotions, as well as emotional and physical changes that occur over time [8]. BID constantly remind young BCS of their painful experience, reduce their self-confidence, initiate or increase their negative emotions, affect their intimate relationships and social interactions, and reduce their quality of life [8, 11].

Self-compassion is a valuable psychological resource for improving BID [12, 13]. Using Buddhist psychology, Neff proposed self-compassion — conceptualized as understanding and accepting one’s pain from a non-judgmental perspective and viewing pain as part of the common human experiences [14]. It encompasses three elements: self-kindness (healing oneself with kindness), common-humanity (recognizing that others are suffering in similar painful experiences), and mindfulness (viewing negative events objectively without ignoring or over-identifying with them) [14]. Self-compassion is a protective factor against BID [15]. Breast asymmetry, scars, and activity limitations that occur during treatment may cause patients to suffer from BID, which is one of the main sources of their negative emotions [16]. Studies have shown that self-compassion activates patients’ self-soothing emotions rather than the escape of emotions arising from negative events [14, 17]. Self-compassion leads to more positive body image and lower body image concerns [17]. When BCS with great levels of self-compassion face these changes in their physical appearance and functional dimensions, they may regard them as a part of the human experience and accept such changes in their bodies more friendly instead of resorting to self-criticism, thus reducing their BID and improving their quality of life [15].

Studies have shown that BID manifested as chronic stress can persist in BCS and affect their quality of life [5, 18]. Coping styles play a central role in dealing with stress, and differ coping styles can independently predict survivors’ long-term psychological function and quality of life [19, 20]. Survivors who adopted adaptive coping strategies exhibit higher psychological well-being and quality of life than those who used maladaptive coping styles [19, 20].

Coping is the process through which individuals attempt to manage the needs arising from stressful events [21]. The main ways of coping include confrontation, avoidance, and acceptance-resignation [22]. Confrontation coping involves actively seeking relevant information and strategies for reducing the influence of stressors. Avoidance coping aims to escape rather than face stress (e.g., denial of the status quo, shifting attention) [22, 23]. Acceptance-resignation coping involves making a greater acquiescence and more concessions for stress. Acceptance-resignation coping is a passive acceptance of stress-producing situations (e.g., resigned, nonresistance) [22]. In this study, to facilitate the interpretation of coping styles, confrontation coping was considered adaptive coping, and avoidance and acceptance-resignation coping were considered maladaptive coping.

Adaptive coping is negatively correlated with BID [24, 25]. As one of the adaptive coping strategies, confrontation coping allows BCS to actively seek effective measures for improving BID (e.g., changing dressing style and exercise habits to improve the appearance and function changes caused by cancer treatment) and reducing negative emotions to improve their quality of life [25]. The use of more maladaptive coping strategies was correlated with greater BID [25, 26]. BCS may struggle to accept body appearance changes caused by cancer treatment, which in turn, may increase their use of avoidance and acceptance-resignation coping strategies, thus leading to lower rates of adaptive coping, higher degrees of psychological distress, and greater BID [25, 26].

Self-compassion is viewed as a key element for coping with stress [27]. Self-compassion has been positively associated with adaptive coping and negatively associated with maladaptive coping [23, 27–29]. Individuals with a high level of self-compassion assess their current situation with an objective attitude and proactively change themselves or their environments appropriately and effectively to cope with their stress. Individuals with low self-compassion are prone to self-blame and over-identification, which could lead them to adopt maladaptive coping (e.g., avoidance or acceptance-resignation coping styles), may aggravate their psychological distress and prolong the duration of their stress [27–29].

Pressure-Cognitive Interaction Theory suggests that cognitive appraisal (i.e., evaluations of personal resources) can indirectly influence mental health outcomes through coping styles [21]. A recent meta-analysis confirmed that self-compassion, as a positive self-evaluation resource, indirectly influenced psychological well-being through coping styles [27]. Beato et al. [28] showed that coping styles mediated the relationship between self-compassion and negative emotional symptoms during coronavirus quarantine phase. Thus, self-compassion may indirectly influence BID through different coping styles.

Overall, BID is a complex and multidimensional concept, and young BCS’ pursuit of beauty in appearance makes their BID more special. BID in young BCS are receiving increasing research attention. Although self-compassion has a positive effect on reducing BID, the potential mechanism is unclear [13]. Coping styles are associated with self-compassion and BID, respectively; thus coping styles may mediate the relationship between self-compassion and BID. This may have positive implications for further understanding the mechanisms underlying self-compassion and BID, and the development of practical and feasible comprehensive interventions for BID.

## Methods

### Aims

**Fig. 1:**
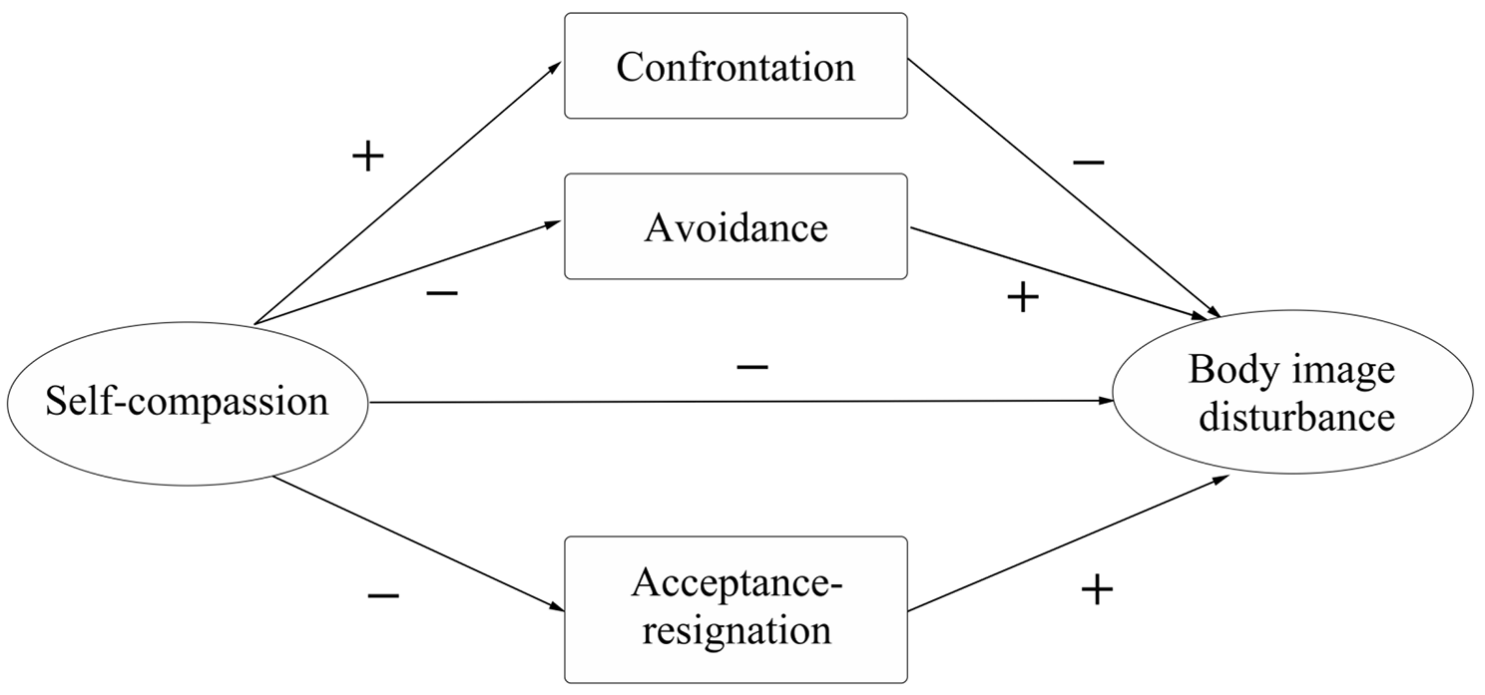
Hypothetical model

The study aimed to investigate the relationship between self-compassion, coping styles, and BID, and assess the different mediating roles of coping styles in the connection between self-compassion and BID among young BCS in China. Furthermore, we proposed the following hypotheses: (H1) In young BCS, the BID is negatively related to self-compassion; (H2) a greater degree of BID is related to less use of confrontation coping and more use of avoidance and acceptance-resignation coping; (H3) greater self-compassion is associated with more use of confrontation coping and less use of avoidance and acceptance-resignation coping; (H4) self-compassion has an indirect effect on BID, which is mediated by this study’s three discussed coping types (Fig. 1).

### Participants and procedures

A cross-sectional design was used to investigate BID, self-compassion, and coping styles in young BCS. Accordingly, from September to December 2021, 310 BCS were recruited through the convenience sampling method in a tertiary cancer hospital in Tianjin, China. The participants were considered eligible if they were (a) diagnosed with breast cancer first; (b) aged from18 to 50 years; (c) received conventional surgical treatment for breast cancer. Furthermore, participants who had co-morbidity with other cancers or were incapable of completing the survey were excluded.

At least 200 cases are deemed necessary to successfully implement the structural equation model [30]. Moreover, according to the sample size estimation formula of structural equation models, the sample size should be 10 to 15 times the number of dimensions in general [31]. This study’s sample size was calculated by 15 times with 16 dimensions. Considering the effective response rate, increase the 20% sample size. Thus, it was determined that the study sample size should exceed 288.

The study obtained the approval from the college Ethics Committee. The researcher provided all participants with the primary study purpose and followed the principles of voluntariness and confidentiality throughout the study. All the participants completed the paper-and-pencil questionnaires after providing written informed consent.

## Measures

### General information

The general information questionnaire included two parts: demographic characteristics (age, marital status, level of education, body mass index, working status, and residence) and clinical characteristics (surgery type, postoperative time, time since diagnosis, and adjuvant therapy).

### Body image disturbance

The Body Image Self-rating Questionnaire for Breast Cancer (BISQ-BC) was used to measure BID in Chinese BCS. Zhou et al. [32, 33] developed and revised the BISQ-BC. The Chinese version of BISQ-BC includes 5 dimensions and 26 items. Answers were rated on 5-point Likert scales from “strongly disagree” to “strongly agree” with summed scores of 26–130. The scale includes seven items about behavioral change, four items about sexual activity change, five items about role change, eight items about psychological change, and two items about social interaction activities change. Higher scores indicated greater BID. Cronbach’s alpha coefficient (α = 0.90) and the structural validity (0.71-0.89) were found to be good [33].

### Self-compassion

The Self-Compassion Scale (SCS) developed by Neff [34] is used for measuring individuals’ level of self-compassion; this study used the version translated to Chinese by Chen et al. [35] It includes 26 items and six subscales (three sets of opposing characteristics): self-kindness (five items)/self-judgment (five items), common humanity (four items)/isolation (four items), and mindfulness (four items)/over-identification (four items). Each item is rated by the subject using a 5-point rating scale (1 = almost never to 5 = almost always). Higher scores reveal greater self-compassion. The Chinese version of SCS reported good reliability, with an internal consistency of 0.84 and test-retest reliability of 0.89 [35].

### Coping styles

The Medical Coping Modes Questionnaire (MCMQ) assessed coping styles. Feifel et al. [22] developed MCMQ to measure individuals’ basic coping strategies concerning stress. Subsequently, it was validated and modified by Shen and Jiang in China [36]. This 20-item Chinese version is grouped into three subscales: confrontation, avoidance, and acceptance–resignation. Responses were recorded using a 4-point Likert scale (from never to all the time). Higher scores represented a greater frequency for participants who adopted certain coping strategies. The internal consistency reliability of each dimension ranged from 0.60 to 0.76 [36].

### Data analysis

Data analysis was performed using SPSS (version 21.0). The variables were described using percentage, mean, and standard deviation (SD). The direction and degree of the relationships among the variances were discussed using Spearman’s correlation analysis. To test the hypothesized mediation model, path analysis was conducted using Mplus 8.0. The Model fit was acceptable if the chi-square/degree of freedom (χ^2^/df) was < 3, the comparative fit index (CFI) and the Tucker–Lewis index (TLI) were ≥ 0.95, the root-mean‐square error of approximation (RMSEA) was < 0.08, and the standardized root mean square residual (SRMR) was < 0.09 [37, 38]. Indirect effects used the samples of 5,000 for bootstrapping, with a 95% confidence interval (CI). An absence of zero in the 95% CI suggested a significant indirect effect.

## Results

### Participant characteristics

A total of 310 patients met the recruitment criteria and 310 completed the paper-and-pencil questionnaires. Table 1 presents the 310 participants’ characteristics for demographic and clinical. Participants’ mean age was 42.64 (SD = 5.79), and 56.1% of them were overweight or obese (mean of body mass index = 24.43, SD = 2.76, range 17.2–34). Modified radical resection was performed in 73.2% of the participants, and 8.1% had received breast-conserving surgery.

The result showed that 50.8% (n = 152) of the participants were disturbed by their body image (median score > 78).

Table 2 shows the scores and internal consistency reliability of each scale.

**Table 1:** Demographic and clinical characteristics (n = 310)

Variable	n (%)
**Marital status**	
Spouse	297 (95.8)
No spouse	13 (4.2)
**Education**	
Junior high school or lower	23 (7.4)
Associate’s degree	108 (34.8)
Senior high school or technical school	88 (28.4)
Bachelor’s degree or above	91 (29.4)
**Employment status**	
Employed	182 (58.7)
Unemployed	128 (41.3)
**Residence**	
City	240 (77.4)
Rural	70 (22.6)
**Time since diagnosis (months)**	
≤ 3	73 (23.5)
4–6	114 (36.8)
7–12	98 (31.6)
>12	25 (8.1)
**Time since surgery (months)**	
≤ 3	109 (35.2)
4–6	111 (35.8)
7–12	70 (22.6)
>12	20 (6.4)
**Adjuvant treatment**	
Chemotherapy	304 (98.1)
Targeted	164 (52.9)
Endocrine	145 (46.8)
Radiotherapy	68 (21.9)
**Surgical procedure**	
Modified radical resection	227 (73.2)
Total mastectomy	52 (16.8)
Breast-conserving surgery	25 (8.1)
Reconstruction	6 (1.9)

**Table 2:** Scores and cronbach’s alpha of each questionnaire (n = 310, M ± SD)

Item	Total score	Cronbach’s alpha
Body image disturbance	78.83 ± 15.48	0.972
Body image-related behavior change	22.55 ± 4.81	0.942
Body image-related sexual activity change	12.31 ± 2.52	0.894
Body image-related role change	14.93 ± 2.88	0.883
Body image-related psychological change	23.87 ± 5.60	0.937
Body image-related social change	5.18 ± 1.34	0.730
**Self-compassion**	**17.84 ± 3.97**	**0.962**
Over identification	12.18 ± 2.96	0.852
Self-kindness	15.20 ± 4.08	0.892
Common humanity	12.02 ± 3.13	0.810
Isolation	11.82 ± 2.52	0.822
Self-judgment	14.72 ± 3.20	0.711
Mindfulness	11.40 ± 3.11	0.799
**Confrontation**	**19.98 ± 3.73**	**0.770**
**Avoidance**	**16.37 ± 3.72**	**0.776**
**Acceptance-resignation**	**10.91 ± 2.74**	**0.772**

### Correlation analysis

Table 3 demonstrates the association of the variables. Lower degrees of self-compassion in young BCS were associated with greater BID (*r* = -.865, *P* < .01). Higher degrees of self-compassion were correlated with higher use of confrontation coping, and lower use of avoidance and acceptance-resignation coping (*r* = .878, *P* < .01; *r* = -0.850, *P* < .01; *r* = -0.885, *P* < .01). Additionally, confrontation coping was negatively correlated with BID in young BCS (*r* = -0.922, *P* < .01), and avoidance and acceptance-resignation coping were positively correlated with BID in young BCS (*r* = .894, *P* < .01; *r* = .913, *P* < .01). The study results thus supported our hypotheses H1, H2, and H3.

**Table 3:** The correlations between body image disturbance, self-compassion, confrontation, avoidance, and acceptance-resignation coping (n = 310, r)

	1	2	3	4	5	6	7	8	9	10	11	12	13	14	15	16
**1. Body image disturbance**	1															
2 .Behavior change	0.908^**^	1														
3. Sexual activity change	0.908^**^	0.799^**^	1													
4. Role change	0.831^**^	0.638^**^	0.761^**^	1												
5. Psychological change	0.953^**^	0.808^**^	0.835^**^	0.763^**^	1											
6. Social change	0.682^**^	0.518^**^	0.556^**^	0.612^**^	0.665^**^	1										
**7. Self-compassion**	**− 0.865** ^**^	− 0.816^**^	− 0.772^**^	− 0.705^**^	− 0.833^**^	− 0.567^**^	1									
8. Self-judgment	− 0.815^**^	− 0.762^**^	− 0.731^**^	− 0.683^**^	− 0.789^**^	− 0.519^**^	0.914^**^	1								
9. Isolation	− 0.724^**^	− 0.685^**^	− 0.648^**^	− 0.625^**^	− 0.702^**^	− 0.436^**^	0.834^**^	0.802^**^	1							
10. Over identification	− 0.765^**^	− 0.713^**^	− 0.711^**^	− 0.655^**^	− 0.726^**^	− 0.528^**^	0.894^**^	0.777^**^	0.746^**^	1						
11. Self-kindness	− 0.784^**^	− 0.761^**^	− 0.684^**^	− 0.613^**^	− 0.750^**^	− 0.517^**^	0.917^**^	0.819^**^	0.662^**^	0.756^**^	1					
12. Common humanity	− 0.798^**^	− 0.762^**^	− 0.693^**^	− 0.643^**^	− 0.765^**^	− 0.549^**^	0.932^**^	0.816^**^	0.732^**^	0.821^**^	0.861^**^	1				
13. Mindfulness	− 0.728^**^	− 0.669^**^	− 0.663^**^	− 0.606^**^	− 0.696^**^	− 0.517^**^	0.867^**^	0.717^**^	0.639^**^	0.782^**^	0.785^**^	0.801^**^	1			
**14. Confrontation**	**− 0.922** ^**^	− 0.843^**^	− 0.844^**^	− 0.746^**^	− 0.890^**^	− 0.634^**^	**0.878** ^**^	0.813^**^	0.724^**^	0.777^**^	0.796^**^	0.817^**^	0.755^**^	1		
**15. Avoidance**	**0.894** ^**^	0.834^**^	0.826^**^	0.701^**^	0.862^**^	0.567^**^	**− 0.850** ^**^	− 0.802^**^	− 0.706^**^	− 0.757^**^	− 0.771^**^	− 0.785^**^	− 0.711^**^	− 0.911^**^	1	
16. **Acceptance-** **Resignation**	**0.913** ^**^	0.836^**^	0.840^**^	0.744^**^	0.880^**^	0.628^**^	**− 0.885** ^**^	− 0.818^**^	− 0.736^**^	− 0.796^**^	− 0.795^**^	− 0.816^**^	− 0.747^**^	− 0.931^**^	0.885^**^	1

^**^P < .01 (2-tails)

### Mediation analysis

**Fig. 2:**
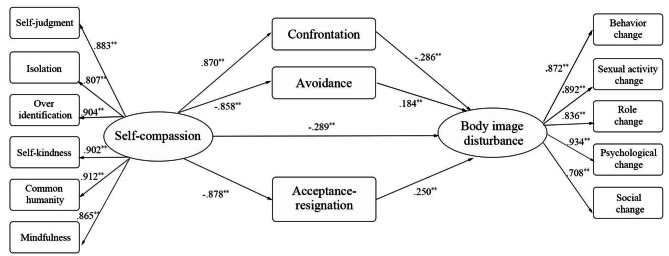
Diagram of the final model: the association between Self-compassion and Body image disturbance ^**^P < .01(2-tails)

The fitting index of the mediation model was good (χ^2^/df = 3.437, CFI = 0.968, TFI = 0.958, NFI = 0.955, RMSEA = 0.089, SRMR = 0.026), as shown in Fig. 2; this result was consistent with our hypothesized model. Self-compassion had significant direct effects on BID (β = -0.289, *P* < .01), confrontation (β = 0.870, *P* < .01), avoidance (β = -0.858, *P* < .01), and acceptance-resignation coping (β = -0.878, p < .01). Confrontation coping directly negatively predicted BID (β = 0.286, *P* < .01), and avoidance (β = -0.184, *P* < .01), and acceptance-resignation coping (β = -0.250, *P* < .01) directly positively predicted BID.

Moreover, 95% CI in the mediation model did not include 0, and all the indirect effects were significant. Self-compassion indirectly affected BID through confrontation (β = -0.248, *P* < 0.001), avoidance (β = -0.158, *P* < 0.046), and acceptance-resignation coping (β = -0.219, *P* < 0.014) (Table 4). Total indirect effects accounted for 68% of the total effects, and the mediation effects of these three paths accounted for 40% (Sc→Co→BID), 25% (Sc→Av→BID), and 35% (Sc→Ar→BID) of the total indirect effects, respectively. Consistent with our hypothesis H4, the above-discussed three coping styles mediated the relation from self-compassion to BID.

**Table 4:** Standardized effect estimation of mediation model

	Effect	95%*CI* Lower	95%*CI* Upper	*P*
Total effect	− 0.915^**^	− 0.966	− 0.849	0.001
Direct effect	− 0.289^**^	− 0.673	− 0.095	0.005
Total indirect effect	− 0.626^**^	− 0.808	− 0.281	0.004
Sc→Co→BID	− 0.248^**^	− 0.587	− 0.096	<0.001
Sc→Av→BID	− 0.158^*^	− 0.438	− 0.003	0.046
Sc→Ar→BID	− 0.219^*^	− 0.485	− 0.054	0.014

Abbreviations: Sc, self-compassion; BID, body image disturbance; Co: confrontation; Av, avoidance; Ar: acceptance-resignation; ^*^P < .05, ^**^P < .01

## Discussion

The study results demonstrated that 50.8% of young BCS had poor body image. In this study only 8.1% of patients underwent breast-conserving surgery and 1.9% of patients underwent reconstructive surgery, and most patients suffered from breast asymmetry, which may be related to the fact that more than half of the participants had BID. Self-compassion, coping styles, and BID were correlated in participants. Additionally, confrontation, avoidance, and acceptance-resignation coping played a role of mediation in the association between self-compassion and BID. This study further identified the key role of coping styles between self-compassion and BID.

### Self-compassion negatively related to BID

Our correlation analysis found that the self-compassion of young BCS was directly and negatively associated with BID, which supported the hypothesis H1. This result confirms previous studies’ findings regarding the association of increasing self-compassion with less BID [12, 14, 39]. Young BCS view changes in their appearance and function as a part of their suffering. Individuals with high levels of self-compassion are prone to practice self-kindness and be less self-critical while avoiding excessive pursuits of perfection; thus, although there may be a gap between their physical appearance and the “ideal” body image, BCS are unlikely to resort to over-denial or debase themselves (e.g., make statements such as “I am a loser”) [16]. Meanwhile, a sense of common humanity and mindfulness can prevent BCS from viewing their painful experiences in isolation and help them to practice appropriate emotional distancing from their painful conditions, accept the BID with self-forgiveness, and try to find effective measures for changing their status quo positively [39]. Thus, a high level of self-compassion is an important resource for managing BID. This suggests that it may be feasible to reduce BID among young BCS through self-compassion in China, oncology nurses should pay more attention to the BID status of young BCS, and improve the self-compassion level of young BCS through structured online writing exercises [15].

### The mediation effect of confrontation coping

This study confirmed that confrontation coping was positively associated with self-compassion, and negatively associated with BID, thus partially supporting hypotheses H2 and H3. Based on the correlation between variables we established a hypothetical model mediated by confrontation coping. The results demonstrated that confrontation coping as a mediator influences the relation between self-compassion and BID, which is consistent with hypotheses H4. The results provided a new basis for formulating comprehensive measures to improve the BID. Young BCS with higher levels of self-compassion are more likely to adopt confrontation coping, resulting in less BID. The mediating effect of confrontation coping was found to be the most significant, which suggested that self-compassion is primarily influenced BID by confronting coping. It is further suggested that confrontation coping can be used as a necessary intervention factor to improve BID in young BCS.

Self-compassion can help BCS reappraise stressful events with a more balanced perspective and make them accepting of their own flaws and inadequacies under the influence of the common humanity style of thinking, thus further reducing the threat of stressors [40]. Self-compassion serves as a positive psychological resource for adaptive coping, which enables patients to adopt confrontation coping in stressful situations. This is consistent with the findings of Neff [23]. Those who adopt confrontation coping are less likely to develop avoidance behaviors, but actively seek social support, improve their appearance, and engage in psychological self-empowerment [25]. The positive changes assist in the return to a steady and healthy state, which in turn improved BID [40].

### The mediation effects of avoidance and acceptance-resignation coping

This result suggested that young BCS with a low level of self-compassion are more likely to use avoidance and acceptance-resignation coping; this could lead them to develop greater BID, confirming the part of hypotheses H2 and H3. Using the hypothesis model, this study verified the mediating role of avoidance and acceptance-resignation coping in self-compassion and BID, thus supporting the part of hypotheses H4.

Under the same stress conditions, BCS with a lower level of self-compassion were likely to engage in excessive self-criticism and self-isolation, indulge in painful events, ignore or avoid the influence of stress events on themselves [40]. Thus, they were more likely to adopt maladaptive coping styles, which affected their BID. Those who adopt acceptance-resignation coping hold a passive and accepting attitude and show indifference and disinterest towards their condition [22]. Some BCS may also adopt avoidance coping for fear of negative comments and glances from others about their changing body image [6]. For example, BCS are more likely to avoid situations that highlight aspects of their physical appearance in order to protect themselves from embarrassment and low self-esteem [25]. Although these coping styles can help individuals alleviate their negative emotions in the short term, in the long run, they can lead to dysfunction, making these individuals addicted to pain and failure, which aggravated their BID and associated psychological distress [27, 39, 41]. Germer et al. [42] showed that training in self-compassion can reduce maladaptive coping and psychological distress. Thus, reducing individual avoidance and acceptance-resignation coping to stressors may be one of the action mechanisms by which self-compassion affects BID.

In the mediation model of this study, nearly two-thirds of the association between self-compassion and BID was explained by coping styles, suggesting that coping styles are important in self-compassion measures to improve BID. Therefore, it is suggested that oncology nurses can develop and implement comprehensive interventions guided by the above pathways. Building self-compassionate for young BCS in coping with stressful events can promote adaptive coping, reduce maladaptive coping, and effectively improve BID.

### Limitations

Although the findings are meaningful, there are several limitations to this study that should be further addressed. First, the cross-sectional design restricted our ability to validate causal relationships for variables. Moreover, the BID was affected by the individuals and their environment; this is a dynamic process, and individual coping styles could also change. Longitudinal research designs are necessary for clarifying the path relationship between self-compassion, coping styles, and BID in young BCS in a dynamic context. Second, the study was conducted in only one hospital, and most of the participants had been diagnosed one year earlier; therefore, the representativeness of the sample was limited. It is suggested that future researchers increase the diversity of their samples and conduct multi-center studies.

## Conclusions

This study validated the mediation effects of different coping styles between self-compassion and BID among young BCS in China, thus providing support for further understanding the mechanism between self-compassion and BID, and implementing comprehensive interventions for BID. We identified confrontation coping as the most significant mediation effect on self-compassion and BID in young BCS. Therefore, in order to improve the intervention effects of BID, oncology nurses should focus on the coping styles of young BCS and promote adaptive coping.

## Data Availability

Data are not made public due to privacy and ethical restrictions. The data for the results of this study will be available from the corresponding author on reasonable request.

## Abbreviations

BCS: Breast cancer survivors
BID: Body image disturbance
χ^2^/df: Chi-square/degree of freedom
CFI: Comparative fit index
TLI: Tucker–Lewis index
RMSEA: Root-mean‐square error of approximation
SRMR: Standardized root mean square residual M:Mean
SD: Standard deviation
CI: Confidence interval
Sc: Self-compassion
Co: Confrontation
Av: Avoidance
Ar: Acceptance-resignation
